# Dual-Scale Polymeric Constructs as Scaffolds for Tissue Engineering

**DOI:** 10.3390/ma4030527

**Published:** 2011-03-01

**Authors:** Carlos Mota, Dario Puppi, Dinuccio Dinucci, Cesare Errico, Paulo Bártolo, Federica Chiellini

**Affiliations:** 1 Laboratory of Bioactive Polymeric Materials for Biomedical and Environmental Applications (BIOlab), Department of Chemistry and Industrial Chemistry, University of Pisa, via Vecchia Livornese 1291, 56010 San Piero a Grado (Pi), Italy; E-Mails: carlos.mota@ns.dcci.unipi.it (C.M.); d.puppi@dcci.unipi.it (D.P.); dinucci@ns.dcci.unipi.it (D.D.); cesare@ns.dcci.unipi.it (C.E.); 2 Centre for Rapid and Sustainable Product Development, Centro Empresarial da Marinha Grande, Rua de Portugal—Zona Industrial, 2430-028 Marinha Grande, Portugal; E-Mail: pbartolo@ipleiria.pt

**Keywords:** additive manufacturing, electrospinning, scaffold, tissue engineering

## Abstract

This research activity was aimed at the development of dual-scale scaffolds consisting of three-dimensional constructs of aligned poly(ε-caprolactone) (PCL) microfilaments and electrospun poly(lactic-*co*-glycolic acid) (PLGA) fibers. PCL constructs composed by layers of parallel microsized filaments (0/90° lay-down pattern), with a diameter of around 365 μm and interfilament distance of around 191 μm, were produced using a melt extrusion-based additive manufacturing technique. PLGA electrospun fibers with a diameter of around 1 μm were collected on top of the PCL constructs with different thicknesses, showing a certain degree of alignment. Cell culture experiments employing the MC3T3 murine preosteoblast cell line showed good cell viability and adhesion on the dual-scale scaffolds. In particular, the influence of electrospun fibers on cell morphology and behavior was evident, as well as in creating a structural bridging for cell colonization in the interfilament gap.

## 1. Introduction

Tissue engineering has been receiving great interest over the last two decades due to its potential in developing living constructs that can meet individual tissue defects. One of the most challenging purposes in this area is to create a biodegradable matrix, commonly referred to as a scaffold, which can provide cells with a three-dimensional (3D) support for the growing tissue. Several polymeric materials and processing methodologies have been investigated for the development of scaffolds with different architecture [1,2,3]. In particular, 3D networks of polymeric structural elements of different geometry and size, such as fibers or filaments with a diameter in the micro and nanoscale, have been proposed as suitable scaffolds due to their high and interconnected porosity as well as high surface area to volume ratio that can promote the adhesion and migration of cells, and the mass transport phenomena associated with cellular activity and material degradation [4,5,6,7,8].

Fused Deposition Modeling (FDM) is a layer-by-layer additive manufacturing technique, based on Computer Aided Design (CAD) and Manufacturing (CAM). It is widely studied for scaffold fabrication thanks to its ability to produce porous polymeric matrices with a reproducible structure, customized external shape and internal morphology [9,10,11]. A layer is obtained by depositing, with a predefined pattern, an extruded filament of a polymer melt and the 3D scaffold is built by putting layers one on top of the other. In this way, the final 3D architecture is determined by the pattern of the deposited filament in each single layer.

A considerable amount of literature has been published in the last years on topographic reaction features of cells in the nanometer range as well as on nanoscale structuring of materials to guide cell behavior [12]. The influence of nanosized elements on the adhesion and activity of different cell lines has been demonstrated by numerous studies [13,14]. For instance the adhesion, proliferation, and alkaline phosphatase activity of osteoblasts have been shown to be significantly affected by nanoscale surface topography [15,16,17,18]. Among other nanofiber fabrication techniques, electrospinning is the most popular and exploited in the field of tissue engineering because of its simplicity, cost effectiveness, and ability to produce polymeric nanofiber assemblies resembling the highly porous structure of native extracellular matrix. This offers the necessary 3D environment for cells to maintain their phenotypic shape and establish natural behavior patterns [19].

Generally, FDM filaments have a diameter of hundreds of micrometers while electrospun fibers display a diameter ranging from few micrometers down to tens of nanometers. A number of studies have shown that 3D structures composed of an assembly of filaments with diameters in the range of hundreds of micrometers enable good *in vitro* cell adhesion and proliferation throughout the whole scaffold. However, it has been highlighted that cells attached to different filaments are hardly able to interact due to the large interfilament distance (hundreds of micrometers) causing limited bridging between cell settlements on different filaments [20,21]. In comparison, electrospun micro/nanometer fiber meshes possess much larger surface area, and as consequence much more binding sites to cell membrane receptors, and pore dimensions (given by the interfiber distance) from nanometers up to few micrometers, allowing for cell adhesion on different fibers. However, the small pore size together with the high fiber packing density can limit cell infiltration in the mesh, as often observed in experimental studies [22,23,24], insomuch that nanofiber constructs have been also proposed as an effective means to prevent post-surgery abdominal adhesion by providing a barrier function [25].

Multi-scale network structures composed of structural elements, in the form of fibers or filaments with different size scales have been developed over the past years in order to improve scaffold architecture. Tuzlakoglu *et al*. [5] were the first to propose this concept by developing scaffolds combining nano- and micro-fibers from starch based biomaterials, produced by means of a two step methodology comprising fiber bonding and electrospinning. They showed that the presence of nanofibers influenced the shape and cytoskeletal organization of human osteoblast-like cells and rat bone marrow stromal cells, and improved their cell viability and alkaline phosphatase production. Similarly, Pham *et al*. [26] developed electrospun poly(ε-caprolactone) (PCL) scaffolds consisting of alternating layers of microfibers and nanofibers. By employing bilayered constructs, they showed that the presence of nanofibers enhanced the spreading of rat marrow stromal cells even if it reduced the infiltration of cells into the scaffolds. As reported in the literature, 3D structures that combine layers of microsized structural elements and nanofibers meshes showed how the presence of electrospun fibers could enhance biological performance [27,28,29,30,31,32].

The present research activity was aimed at the development of dual-scale structures in order to couple the mechanical strength and structural reproducibility of 3D microfilament constructs fabricated by additive manufacturing techniques to the advantage of electrospun ultrafine fibers in enhancing cell interaction with polymeric materials. Two different materials were employed for the fabrication of the bioextrusion (BE) structure (*i.e*., PCL) and the electrospun fibers (*i.e*., poly(lactic-*co*-glycolic acid), PLGA). 3D PCL constructs were produced by means of an additive manufacturing BioExtruder technology that comprises two different deposition systems for the fabrication of multimaterial scaffolds incorporating cells and bioactive agents [20]. During the experimental work, the processing conditions for the collection of PLGA fibrous meshes on top of microfilament constructs were investigated and correlated to the morphology of the resulting dual-scale scaffolds by means of scanning electron microscopy (SEM). In order to evaluate the *in vitro* cytocompatibility of the developed scaffolds, MC3T3 murine preosteoblast cells were seeded and cultured onto BE and dual-scale structures. Cell response, in terms of viability, proliferation and morphology, to the prepared tissue engineered constructs was investigated by tetrazolium salts (WST-1 cell proliferation reagent) and confocal laser scanning microscopy (CLSM).

## 2. Results and Discussion 

The influence of scaffold composition, topography and architecture on cell behavior has been highlighted over the past years by various studies that have shown how they determine the mechanism and sites’ geometry of cell adhesion, affecting the degree of spreading and cytoskeleton orientation of cells [13]. In addition, it has also been highlighted how scaffold composition influences the mass transport phenomena within the scaffold, regulating oxygen and nutrients transport, as well as mechanical stresses acting on cells [33].

The present research activity was focused on the development of a dual-scale tissue engineered scaffold by coupling electrospun ultrafine fibers to microfilament structures; this allows for the introduction of a nanoscale topography that offers high surface area for cell adhesion, as well as a structural bridging between adjacent microfilaments that can create a microenvironment favoring cell mobility and interaction.

### 2.1. Development of Dual-Scale Scaffolds

**Figure 1:**
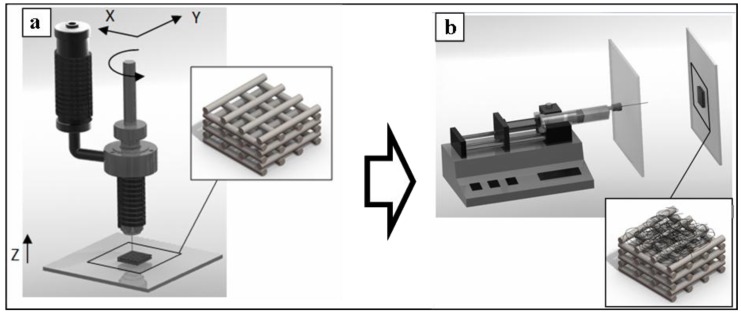
Scheme of the two-step process for the fabrication of 3D dual-scale scaffolds: (**a**) firstly, a 3D microfilament structure of poly(ε-caprolactone) (PCL) was built up layer-by-layer by a bioextrusion (BE) process; (**b**) secondly, electrospun poly(lactic-*co*-glycolic acid) (PLGA) ultrafine fibers were collected on the top of the PCL structures.

Dual-scale scaffolds were produced by collecting PLGA ultrafine fibers on the top of 3D PCL microfilament constructs (see Section 3.2). First, PCL structures were obtained by a layer-by-layer approach using a BE process (Figure 1(a)), then PLGA was electrospun on top of PCL structures employing a screen-to-screen electrospinning configuration (Figure 1(b)).

#### 2.1.1. Fabrication and Morphological Characterization of 3D PCL Structures

**Figure 2:**
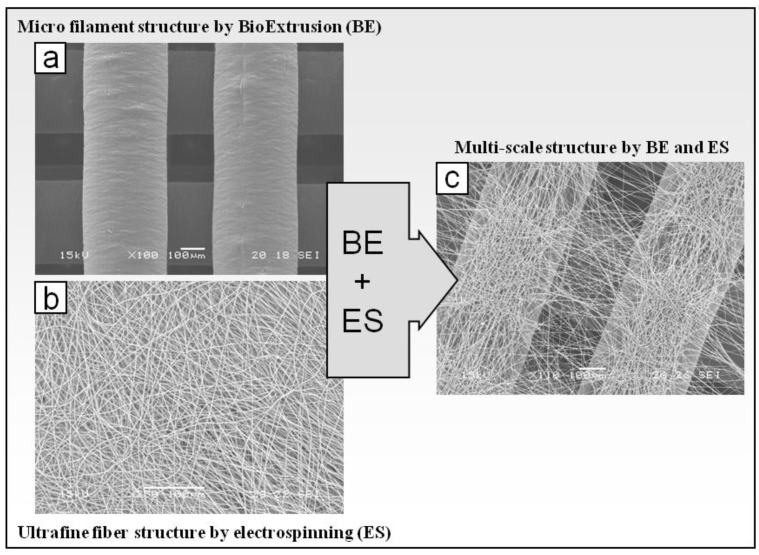
Scanning electron microscopy (SEM) micrographs of (**a**) microsized PCL structure produced by BE system; (**b**) ultrafine PLGA fibers produced by electrospinning (ES); **(c**) dual-scale structure composed of PCL microfilaments and PLGA ultrafine fibers.

To fabricate microsized filaments, melted polymer was extruded through the nozzle translating with a predefined 0/90º lay-down pattern. In this way, 3D architectures were built up by overlapping layers composed of parallel filaments such that the filaments of adjacent layers were perpendicular. Scaffolds with different thickness size, depending on the number of layers (4 to 12 layers), were produced. As shown by SEM analysis (Figure 2(a)), 3D PCL constructs were composed by layers of parallel microsized filaments with an average diameter of 365.4 ± 18.3 μm and interfilament distance of 190.8 ± 21.8 μm.

Over the past decade, and since the first work reported by Hutmacher [1] on scaffolds fabricated by FDM technology, a number of studies have been published on melt extrusion-based additive manufacturing techniques for application in tissue engineering [34,35,36,37]. Such techniques are suitable for the production of scaffolds with highly reproducible morphology, and tuneable pore architecture, size and interconnectivity. A study by Zein *et al.* [38] on PCL scaffold produced by FDM demonstrated a correlation between scaffold architecture (*i.e.*, porosity, lay-down pattern and filament alignment) and compressive mechanical properties. By investigating various lay down patterns, they concluded that 0/90° offers the best mechanical outcome in terms of compression strength. The idea of the present investigation was thus to combine the fabricated 0/90° aligned microfilament scaffolds with electrospun fibers in order to develop a dual-scale structured scaffold with optimized mechanical and biological performances.

#### 2.1.2. Fabrication and Morphological Characterization of Dual-Scale Scaffolds

The experimental conditions for the fabrication of dual-scale scaffolds by means of PLGA electrospinning onto the top of PCL matrices (Figure 1(b)) were investigated. The electrospinning parameters for obtaining uniform PLGA fibers (C = 20% w/v, V = 25 kV, d_1_ = 15 cm, d_2_ = 18 cm, F = 2 mL h^−1^), optimized in previous related work [39], were applied. The influence of the processing parameters, such as the thickness of the 3D structure and the processing time, on the morphology of the electrospun fiber assembly was investigated.

**Figure 3:**
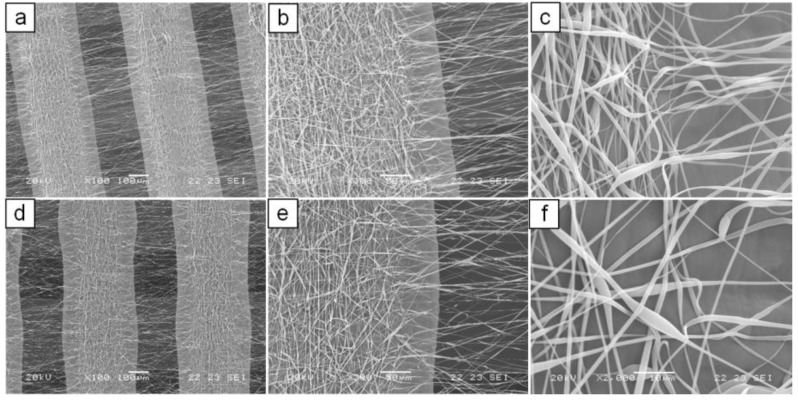
SEM micrographs of dual-scale structures obtained using a screen-to-screen configuration with electrically insulated needle. (**a**), (**b**), (**c**), 4 layers structure; (**d**), (**e**), (**f**) 12 layers structure.

As shown in Figure 2(c), electrospun fibers collected onto PCL matrices presented a certain degree of alignment instead of the typical randomly oriented fiber assembly (Figure 2(b)) obtained with a conventional electrospinning apparatus. In particular, a two-direction alignment orientation was observed: fibers collected onto microfilaments appeared aligned along the microfilament axis while fibers bridging two microfilaments were nearly perpendicular to the microfilament axis. However, the fabrication process suffered from lack of reproducibility in terms of morphology, likely because of perturbation of the electric field at the collection area. To overcome this disadvantage, an electrical insulation was applied to the metallic needle, which has shown to narrow the electrospinning collection area [40]. With this processing configuration and by applying the optimized electrospinning conditions (d_1_ = 14 cm, d_3_ = 7 cm, V = 28 kV) the reliability and reproducibility of the fabrication process was improved and a better control over the packing density of the collected fibers was achieved. Dual-scale structures with different thickness (4–12 layers) showing alignment of electrospun fibers in the gap between microfilaments were produced (Figure 3). Moreover, by optimizing the processing time (60 s) and solution feed rate (0.5 mL h^−1^), electrospun layers with low fiber packing density and average fiber diameters of around 1 μm (0.81 ± 0.40 μm for 4 layers, 1.04 ± 0.39 μm for 8 layers, 1.11 ± 0.58 μm for 12 layers; data not statistically different for p < 0.05) were obtained (Figure 3(c), (f)). Such processing conditions were selected for the production of scaffolds that were further characterized.

Various works have shown how acting on electric field parameters, by changing the spinneret and/or the collection system, can influence the morphology and assembly of electrospun fibers. Teo and Ramakrishna [41] recently reviewed the various methods proposed to obtain fibers alignment by changing the collection system, such as rotating cylinder [42,43,44,45] and disk [46,47], dual ring configuration [48], and AC potential [49]. For instance, Li *et al.* [50,51] showed how it is possible to obtain uniaxially aligned electrospun fibers by employing two parallel strips of electrical conductive materials as collection counter-electrode system. They explained the alignment mechanism with the influence of the insulating gap between strips on the electric field structure and, as consequence, of the direction of the electrostatic forces acting on a fiber that is sitting across the gap. The electrostatic interactions between the charged depositing fibers and the strips should simultaneously pull the fiber towards the edges of the two electrodes leading to its uniaxial alignment across the gap. In addition, Zhang and Chang [52] investigated alignment of electrospun polymer fibers collected onto a patterned collector composed of parallel wires made from electroconductive materials. In addition to fiber alignment in the gap, a certain degree of orientation for fibers collected onto wires with a diameter of few hundreds of micrometers was observed. Such a phenomenon was explained by the electrostatic interaction between the charged fibers and the opposite charges that they induce on the wire surface. Hence, regarding the present study, it could conceivably be hypothesized that the presence of aligned polymer filaments onto the collector led to electrostatic interactions between the depositing fiber and the polarized filaments, similar to those hypothesized in the above reported studies causing a two direction alignment of fibers collected onto filaments and in the gap.

Although electrospun fibrous meshes have raised great interest in the tissue engineering field, one of their main disadvantages is the small pore size limiting cell infiltration inside the mesh [25]. The low packing density of the electrospun layer morphology optimized in the present work is characterized by interfiber distances likely suitable for cell migration in the inner part of the scaffold.

In addition, the employment of two different materials (*i.e*., PCL and PLGA) can provide enhanced control over degradation kinetics of the dual-scale scaffold. Indeed, PLGA generally degrades in physiological conditions at a faster rate than PCL, and electrospun fibers (~1 μm diameter) have much larger specific surface area than BE filaments (~360 μm diameter), which can greatly accelerate the mass transport phenomena involved in material degradation. It can thus be assumed that PLGA fibers will be degraded much earlier than BE structure. It would be interesting to tune the degradation rate of PLGA fibers (e.g., changing the fiber diameter, the LA/GA molar ratio or the molecular weight) to match the formation rate of the growing tissue in the interfilament gap, in order to provide cells with the necessary support for adhesion and proliferation for sufficient time. In addition, such great differences in surface area and degradation rate can be exploited to endow the scaffold with advanced drug release capability by loading fibers with one or more bioactive agents. This strategy could lead to multi-step kinetics and/or multi-drug release systems.

### 2.2. Biological Results

Quantitative viability and proliferation assay (WST-1) and morphology analysis (confocal laser microscopy, CLSM) were carried out on either BE or dual-scale tissue engineered constructs, with three different thicknesses (4, 8 and 12 layers), after seven days of culture by using the MC3T3 murine preosteoblast cell line.

**Figure 4:**
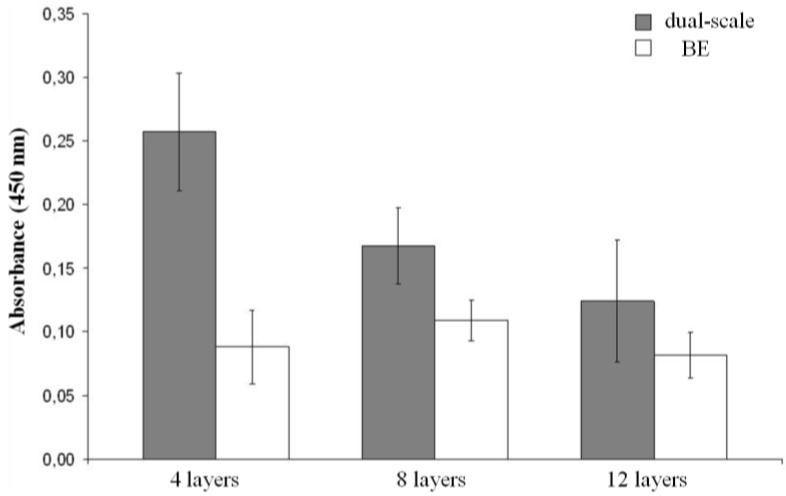
Viability histograms of the MC3T3 cell line after 7 days of culture using either BE or dual-scale tissue engineered constructs.

The values obtained from WST-1 assay were elaborated and are shown in Figure 4. After seven days of cell culturing, significantly higher mitochondrial dehydrogenase activity was observed in dual-scale when compared to BE scaffolds. Regarding the 4 layer scaffolds, BE structures exhibited a cellular viability three times lower than dual-scale analogous scaffolds. However, by increasing the scaffold thickness the cell viability of dual scale constructs decreased while that of BE constructs did not vary significantly, resulting in smaller differences in viability between the two types of scaffolds (Figure 4).

**Figure 5:**
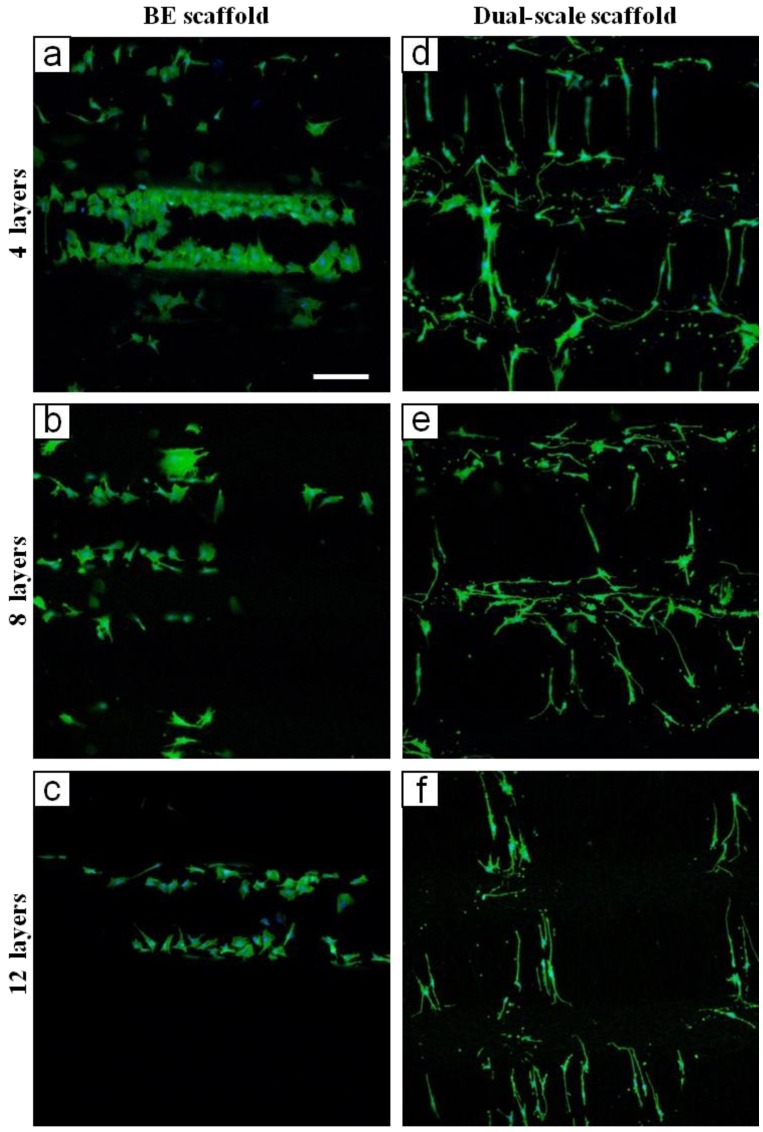
CLSM micrographs of MC3T3 cells grown on BE scaffolds (**a**)–(**c**) and dual-scale scaffolds (**d**)–(**f**) of different thicknesses. The scale bar of 200 μm in (a) is applicable to all micrographs.

**Figure 6:**
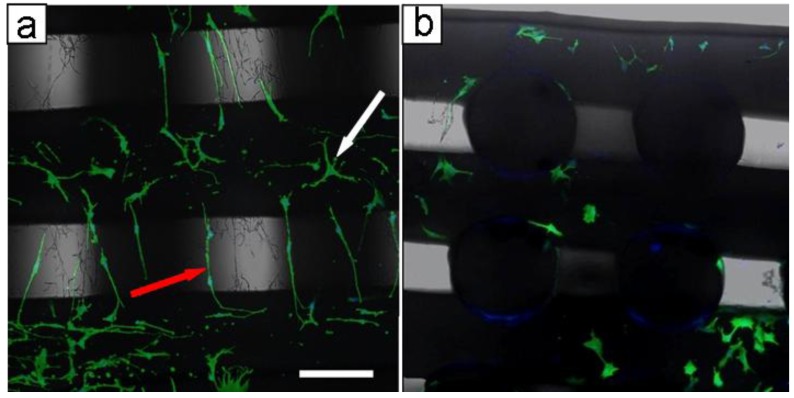
CLSM micrographs of MC3T3 cells grown on dual-scale scaffold after 7 days of culture. (**a**) top view showing difference in morphology between cells adhered on PLGA fibers onto filaments (white arrow) and those adhered on PLGA fibers aligned in the gap (red arrow); (**b**) cross section of dual-scale constructs showing cells grown in inner parts of scaffold. Scale bar of 150 μm in (a) is applicable to (b).

CLSM analysis was performed in order to evaluate the morphology of MC3T3 cells after seven days of culture on BE and dual-scale scaffolds. Cytoskeletal stain demonstrated impressive differences in cell distribution and morphology between the BE and dual-scale constructs (Figure 5). In BE constructs, cells appeared to adhere on PCL filaments (Figure 5(a)–(c)) throughout BE scaffolds. In dual-scale constructs, cells were detected widely on the electrospun layer (Figure 5(d)–(f)) but also in the inner part of the construct (Figure 6(b)). Cells grown onto dual-scale scaffolds exhibited a high number of membrane extroflections and a strong elongated shape, spread along PLGA fibers that act as guidance for the cytoskeleton. On the contrary, cells grown onto BE constructs showed a wide spread morphology mostly related to the typical 2D culture. Fluorescent images revealed a considerable amount of cells evidently adhered and suspended on the PLGA electrospun fibers, in-between two PCL filaments, resulting, as previously shown in SEM micrographs (Figure 3), as a “micro-loom”. Different cell morphologies were observed due to the difference in orientation between PLGA fibers collected onto PCL filaments and those in the interfilament gap. Cells grown onto fibers collected above the PCL filaments showed a star-shaped soma, and long thin extroflections and filopodia with variable length (white arrow in Figure 6(a)); cells grown onto fibers aligned in the gap between PCL filaments showed highly extended cytoskeleton with fewer filopodia (red arrow in Figure 6(a)).

As previously stated, in the last few years some studies have proposed multi-scale scaffolds showing how the nano/microsized ultrafine structural elements can enhance cell adhesion and behavior [5,27,28,29,30,31]. Accordingly, the present study shows that, differently to what is observed in BE structures, cells were able to colonize the interfilament gap when grown in dual-scale constructs. Such a phenomenon was due to the effect of the guidance in cellular adhesion exerted by electrospun fibers, which was also responsible for the slight morphology differences between cells adhered on fibers with different orientation. The cytoskeletal organization features of cells adhered on fibers collected onto microfilaments (white arrow in Figure 6(a)) are consistent with what was observed by other investigations on changes of MC3T3 morphology during differentiation [53]. Moreover, such dual-scale structure is characterized by fiber alignment and high spatial connectivity that is capable to direct cell orientation and migration, supporting previous research on the effects of scaffold structure and fiber alignment on cell behavior [13].

Since from microscopy observations there were no evident differences in the amount of cells grown onto the electrospun layers of the three thicknesses of dual-scale scaffolds, the spread in viability values, evaluated by means of WST-1 assay, is likely due to the difference in cell growth into the inner part of the scaffold. From these results, it is evident that the electrospun layer together with scaffold thickness greatly influence cell adhesion and proliferation. It could be hypothesized that electrospun fibers in the gap exert a sieving action during cell seeding, and in such a way slowing cell diffusion throughout the scaffold and influencing a more uniform cell distribution. This effect may be, however, diminished in thicker scaffolds where cells are dispersed in a larger volume and are not able to cluster and concentrate in areas small enough to allow cell-cell interaction. This could in part explain the attained cell viability results.

## 3. Experimental Section

### 3.1. Materials 

Poly(ε-caprolactone) (PCL, CAPA 6500, Mw = 50,000) was bought from Perstorp Caprolactones Limited (Cheshire, UK), poly(lactic-*co*-glycolic acid) (PLGA, 75:25 lactide/glycolide, Mw = 120,000) from Lakeshore Biomaterials Inc. (Birmingham, USA). All the solvents and chemical reagents were purchased from Sigma-Aldrich (Italy) and used as received.

### 3.2. Fabrication of Dual-Scale Fibrous Scaffolds

Dual-scale scaffolds were produced by electrospinning PLGA solutions on the top of 3D PCL matrices fabricated by a novel BioExtruder device [20,37] (Figure 1).

#### 3.2.1. Production of 3D Scaffolds by Bioextrusion

The BE system is composed by a screw extrusion head equipped with a micronozzle (gauge 23, I.D. = 340 μm) to produce a melt filament from polymer in the form of pellets or powder. The X, Y movement of the micronozzle and the Z movement of the construction table allow the production of 3D structures in a layer-by-layer fashion. The material was melted in the extrusion head reservoir at a temperature of 80 °C and pressurized at 6 bar. The screw extruder was settled at a rotation of 20 r.p.m in order to achieve an accurate deposition and filament uniformity to the employed scanning velocity (20 mm s^−1^). The 3D geometry, defined by means of CAD software, was processed using Matlab (The MathWorks, Inc.) algorithm to calculate the BE deposition pattern. The pattern was defined with X and Y axis increment between parallel filaments of 550 μm and a Z axis interlayer increment of 300 μm. The calculated deposition pattern was converted automatically to an ISO Computer Numerical Control (CNC) language code and processed in the BE device. During the present study, rectangular prism-shaped PCL scaffolds with the base measuring 30 × 30 mm and different thicknesses in dependence on the total number of layers (4–12 layers) were produced.

#### 3.2.2. Electrospinning of Polymer Solution

PLGA was dissolved in acetone at room temperature under gentle stirring for 2 h to obtain a homogeneous solution at a concentration (C) of 20% w/v. For the production of electrospun collection we employed the experimental conditions optimized during a previous related work on electrospinning of PLGA solutions [39]. The electrospinning apparatus consisted of a 10 mL syringe equipped with a 21-gauge blunt needle, a syringe pump (BSP-99 M, Braintree Scientific Inc., Braintree, MA) and two high voltage power supplies of opposite polarity (Spellman High Voltage, UK). To obtain focused fiber collections, we employed a screen-to-screen electrode configuration composed of two parallel metallic screens at different electrical potentials, one functioning as a fiber collector and the other one as an auxiliary electrode. The fluid was fed at a given flow rate to the metallic needle jutting out of a hole in the middle of one plate and the fibers were collected onto the other plate [54]. A sample of PCL scaffold by BE with a base size of 15 × 15 mm was fixed in the central part of the collecting plate to obtain the deposition of electrospun fibers onto its top surface. The distance between the two screens (d_1_) was 18 cm, that between the needle tip and the collector (d_2_) was 15 cm and the applied potential difference (V) was 30 kV; different solution feed rates (F = 0.5–4 mL h^−1^) and various processing times (t = 20–60 s) were tested. In addition, in order to obtain a more uniform electric field, an electrical insulation was applied to the metallic needle by means of a capillary glass tube. In this case, the optimized processing conditions were: d_1_ = 14 cm, distance from the tip of the glass capillary to the collector (d_3_) = 7 cm, V = 28 kV, F = 0.5 mL h^−1^ and t = 60 s. After electrospinning, the samples were first dried under a fume hood and then kept under vacuum for 24 h.

### 3.3. Morphological Characterization

Samples from polymeric scaffolds were cut, gold sputtered and observed by scanning electron microscopy (SEM; Jeol LSM 5600LV, Japan). Average diameter of electrospun fibers and BE filaments, and interfilament distances were determined by means of ImageJ 1.43u software on SEM micrographs (100× magnification for filaments and 2000× for electrospun fibers). The data were calculated over 30 measurements per specimen, taken from randomly selected fields.

### 3.4. Biological Evaluation

#### 3.4.1. Cell Culture onto PCL Scaffolds

Mouse calvaria-derived preosteoblastic cells MC3T3 were obtained from the American Type Culture Collection (ATCC CRL-2593). Cells were cultured as monolayers in Alpha Minimum Essential Medium (alpha-MEM) added with ribonucleosides, 2 mM L-glutamine, 1 mM sodium pyruvate, supplemented with 10% fetal bovine serum and 100 U/mL:100 µg/mL penicillin: streptomycin (GIBCO, Invitrogen Corporation). Cell culturing was carried out in appropriate growing conditions (in a humidified incubator at 37 °C and 5% CO_2_ enriched atmosphere). Prior to seeding, all scaffold were sterilized in a 70% ethanol/water solution dipping for 24 hours then extensively washed with PBS 0.01 M pH 7.4 before exposing to UV light for 40 minutes. To facilitate cell attachment, the scaffolds were left overnight in 2 mL of culture medium in appropriate growing conditions. Subsequently culture, medium was replaced with 1 mL of culture medium containing 1 × 10^4^ cells that was added dropwise directly onto each scaffold placed in a 12 well plate. After 1 h of incubation, 1 mL of culture medium was added to each well. Seeded scaffolds were cultured in appropriated growing conditions for 7 days replacing the culture medium every 3 days. At the end of the culture time samples were processed for microscopy and viability tests.

#### 3.4.2. WST-1 Cell Proliferation Assay

Cell viability and proliferation was measured by using the (4-[3-(4-Iodophenyl)-2-(4-nitrophenyl)-2H-5tetrazolio]-1,3-benzene disulfonate) (WST-1) assay (Roche Molecular Biochemicals). The assay, based on the mitochondrial enzymatic conversion of the tetrazolium salt WST-1 into the soluble and colored formazan salt, was performed after 7 days of culture by incubating living cells with the WST-1 reagent diluted 1:10 for 4 hours at 37 °C. Plates were then analyzed with a Biorad Microplate Reader (Biorad, Hercules, USA). Formazan dye quantity absorbance was measured at 450 nm with the reference wavelength at 620 nm.

#### 3.4.3. Confocal Laser Scanning Microscopy (CLSM)

At culture end-point, scaffolds were rinsed with PBS to remove growing medium and impurities before fixation with 4% paraformaldehyde in PBS 0.01 M pH 7.4 for 45 minutes. Every step of scaffold processing for microscopy stain was performed at room temperature on an orbital plate under mild stirring conditions in order to enhance access of fixative and other solutions through the meshes. Scaffolds were rinsed with PBS and incubated with a PBS solution of Triton X-100 (Sigma-Aldrich, Italy) at 2% volume for 15 minutes to permeabilize cells before incubation with dye solution. PBS solution of 4’-6’-diamidino-2-phenylindole (DAPI; Invitrogen, Italy) and phalloidin-Alexa488 (Invitrogen, Italy) was subsequently added for 1 hour. After dyeing, incubation samples were extensively washed with PBS and placed between two glass coverslips on a microscope stage for observation. A Nikon Eclipse TE2000 inverted microscope equipped with an EZ-C1 confocal laser and D-Eclipse C1-si spectral optical system (Nikon, Japan) was used to analyze the samples. A 405 nm laser diode (405 nm emission) and Argon ion laser (488 nm emission) were employed to excite DAPI and phalloidine fluorophores, respectively. Images were acquired with Nikon EZ-C1 software with identical settings for each sample. Merge images were processed with ACT-2U software (Nikon, Japan).

### 3.5. Statistical Evaluation 

Quantitative data were presented as mean ± standard deviation (SD). Data sets were screened by one-way ANOVA and a Tukey test was used for *post hoc* analysis; significance was defined at p < 0.05.

## 4. Conclusions

The main goal attained during the present research was the combination of BE and electrospinning techniques in order to fabricate dual-scale scaffolds made of two different polymeric materials. This allows coupling the mechanical strength and structural reproducibility of BE constructs with the biological advantages of electrospun fibers in influencing cell behavior. Moreover, the employment of two different polymers can represent a potential means for achieving a better control over degradation kinetics and release rate of loaded bioactive agents. The developed procedure comprises first the fabrication by BE of a PCL construct, consisting of 0/90° patterned microfilaments, and then electrospinning PLGA fibers on top of them. The resulting structure is characterized by electrospun fibers, showing a certain degree of alignment and low packing density, collected in the gap between microfilaments. This structural bridging enables cell colonization of the gap due to cell adhesion at seeding time and/or subsequent cell migration. Moreover, biological investigations highlighted the differences in morphology among cells adhered on microfilaments, on PLGA fibers above microfilaments, and on aligned fibers in the gap, strongly suggesting the capability of the electrospun layer to definitely influence cell response.

Future works will be addressed to the automation of the fabrication technology by means of a system integrating additive manufacturing extrusion-based technique and electrospinning, which was purposely developed and is currently under validation. Such a system will allow structural gradients throughout the construct, in terms of structural element size, geometry and material, to be obtained. A further biological investigation could be focused on the optimization of cell culture conditions as well as on the characterization of cell populations in terms of expression of differentiation markers.
